# KeyPathwayMineR: *De Novo* Pathway Enrichment in the R Ecosystem

**DOI:** 10.3389/fgene.2021.812853

**Published:** 2022-01-31

**Authors:** Konstantinos Mechteridis, Michael Lauber, Jan Baumbach, Markus List

**Affiliations:** ^1^ Chair of Experimental Bioinformatics, TUM School of Life Sciences, Technical University of Munich, Munich, Germany; ^2^ Chair of Computational Systems Biology, University of Hamburg, Hamburg, Germany; ^3^ Department of Mathematics and Computer Science, Faculty of Science, University of Southern Denmark, Odense, Denmark

**Keywords:** pathway enrichment, network analysis, data integration, systems biology, R package

## Abstract

*De novo* pathway enrichment is a systems biology approach in which OMICS data are projected onto a molecular interaction network to identify subnetworks representing condition-specific functional modules and molecular pathways. Compared to classical pathway enrichment analysis methods, *de novo* pathway enrichment is not limited to predefined lists of pathways from (curated) databases and thus particularly suited for discovering novel disease mechanisms. While several tools have been proposed for pathway enrichment, the integration of *de novo* pathway enrichment in end-to-end OMICS analysis workflows in the R programming language is currently limited to a single tool. To close this gap, we have implemented an R package KeyPathwayMineR (KPM-R). The package extends the features and usability of existing versions of KeyPathwayMiner by leveraging the power, flexibility and versatility of R and by providing various novel functionalities for performing data preparation, visualization, and comparison. In addition, thanks to its interoperability with a plethora of existing R packages in e.g., Bioconductor, CRAN, and GitHub, KPM-R allows carrying out the initial preparation of the datasets and to meaningfully interpret the extracted subnetworks. To demonstrate the package’s potential, KPM-R was applied to bulk RNA-Seq data of nasopharyngeal swabs from SARS-CoV-2 infected individuals, and on single cell RNA-Seq data of aging mice tissue from the *Tabula Muris Senis* atlas.

## 1 Introduction

Given the ever-increasing amount of publicly available OMICS data and the expanding size and variety of biological networks, the analysis of these data has become an essential building block of bioinformatics. The combined investigation of networks and OMICS data can provide meaningful insights into the biological background of the studied condition, such as in the discovery of biomarkers, new biological functions, and disease mechanisms (Reimand et al., 2019, p. 482).

The purpose of *de novo* pathway enrichment is to find pathways within a biological interaction network related to phenotypes of interest investigated in case-control OMICS studies. These pathways are extracted as subnetworks containing a high number of biological entities differentially regulated in OMICS data. The advantage of this method is that it detects in addition to known, also novel pathways which are not detected in traditional methods (Alcaraz et al., 2014; Alcaraz et al., 2016).

*De novo* pathway enrichment, as a downstream analysis task, typically depends on processed OMICS and biological interaction networks. OMICS datasets include for instance, gene expression values, DNA methylation signals or single nucleotide variants which can come from primary data or can be obtained through online databases and repositories, while biological interaction networks can be derived from protein-protein-interaction databases or gene regulatory networks. The R statistical framework with its rich package ecosystem (R Core Team, 2020) and its Bioconductor repository allows for an easy retrieval for this kind of data. Moreover, thousands of R packages exist in CRAN, Bioconductor, and GitHub, which provide functionalities in the acquisition, preparation, normalization, and visualization of the datasets. While several tools have been proposed for pathway enrichment (Batra et al., 2017), the integration of *de novo* pathway enrichment in end-to-end OMICS analysis workflows in R is to our knowledge currently limited to BioNet (Beisser et al., 2010). *De novo* pathway enrichment methods generally vary in their performance, depending on the dataset and problem setting selected (Batra et al., 2017). A complementary pathway enrichment tool, KeyPathwayMiner (KPM) (List et al., 2016), is currently only available as a Cytoscape app, a standalone Java application or *via* a web server, making its integration into R-based workflows challenging for typical R users who often lack experience with lower level programming languages and the use of the command line interface. Moreover, the preparation of the suitable input files for KPM from primary data, e.g., a network in SIF format and an indicator matrix of features *vs* samples, is not trivial for most users.

To close this gap, we have implemented an R package KeyPathwayMineR (KPM-R) with extended features and improved usability. To make the integration of KPM-R straightforward, we implemented a multitude of convenience functions. For example, the package’s methods facilitate the retrieval of biological interaction networks from BioGRID (Oughtred et al., 2018) for various organisms. In our vignette we show how OMICS data from repositories like GEO (Davis and Meltzer, 2007a) or TCGA (Colaprico et al., 2016) can easily be downloaded and processed internally into a suitable input format for KPM-R. We further added support for the analysis of single cell RNA sequencing data where users can provide a single-cell RNA-seq object and can pick the comparison to be made. KPM-R allows switching between a local and remote execution depending on the user’s needs. If the user lacks sufficient computing power, the analysis can be run through the provided RESTful service on the KPM web server. The user can also conveniently define a parameter range to simplify the otherwise tedious hyperparameter optimization. KPM-R visualizes the results across hyperparameters to allow determining the most appropriate choice. For the visualization of the extracted *de novo* pathways, we implemented an interactive web app using the R shiny framework. This allows users to browse, visualize and export the sub-networks as an image or machine-readable text files. In contrast to the existing web application (https://keypathwayminer.compbio.sdu.dk/keypathwayminer/), the shiny app can also be used to visualize results that were created offline and serves as a template for users that want to embed KPM-R in more complex shiny apps for data analysis. The vignette also offers guidance of further downstream analysis of the selected pathways which includes gene ontology and gene set enrichment analysis.

To demonstrate the benefits of KPM-R for assembling R data analysis workflows, we used a large dataset of nasopharyngeal swabs from SARS-CoV-2 infected individuals and negative control cases (GSE152075) (Lieberman et al., 2020). Furthermore, we investigated celltype-specific subnetworks in primary mouse tissue from single cell RNA-seq data from the *Tabula Muris Senis* Atlas (Almanzar et al., 2020). In both application cases, we show how the package assists in processing raw data, finding *de novo* pathways relevant to the studied diseases and conducting downstream analyses that offer a more in-depth understanding of the disease mechanisms.

## 2 Materials and Methods

### 2.1 Strategies for Network Extraction

The objective of KPM is to extract maximally connected subnetworks that are enriched in differentially active nodes which can represent e.g., differentially expressed genes. The user can define which nodes are considered active in each case or sample by supplying an indicator matrix. The rows of the matrix describe the nodes (typically genes or proteins) and the columns the samples (see Subsection 2.3.1 for more information on indicator matrices) (Alcaraz et al., 2016, p. 1, p. 4). The task of finding the maximal subnetwork can be defined as a graph-theoretical problem:

Let graph *G* = (*V*, *E*) represent an undirected biological network that consists of a set *V* representing biological entities and a set *E* containing the interactions between them. Further, we have one or, in case of multi-omics data, multiple indicator matrices *A*_1_, …, *A*_*p*_ , e.g., an indicator matrix from a proteomics experiment and another one from a transcriptomics study. The indicator matrices consist of *n* features (e.g., genes) and *m* samples (e.g., patients). These matrices will be combined to a single indicator matrix *A* ^*n*,*m*^. To combine the indicator matrices, the biological entities should be identical for all matrices and the number of samples should be equal. Additionally, we have a mapping *Z*: *V* → {0,1}^*m*^ from each node *v* ∈ *V* to its corresponding row in the matrix *A*. The objective is to extract a subnetwork *G*′ = (*V*′, *E*′) ⊂ *G* such that *G*′ is connected and the set of nodes *V*′ satisfy the constraints of the INES or the GloNE strategy (Alcaraz et al., 2020, p. 183-184).

#### 2.1.1 Individual Node Exceptions Strategy

The INEs strategy uses two parameters *K* and *L* to constrain how maximally connected subnetworks enriched with differentially active nodes are extracted. The *L* parameter describes the maximal number of allowed case exceptions for a particular node in the extracted network (i.e., in an active node, the number of patients/samples in which the corresponding entity (e.g., gene or protein) is not differentially regulated must be less or equal to *L*). *K* is the number of allowed inactive nodes in the extracted subnetwork (i.e., they exceed the number of allowed case exceptions). Intuitively, *L* regulates which genes are considered relevant whereas *K* allows KPM to combine smaller solutions into larger ones using up to *K* genes as connectors. Due to their central role in the subnetwork such nodes may be important for a pathway even if they are not differentially expressed or regulated themselves (Baumbach et al., 2012, p. 1).

Formally, this can be described in the following way: Given a biological network *G* = (*V*, *E*) , an indicator matrix *A* ^*n*,*m*^, *K* node exceptions, *L* case exceptions and *s* being a sample from the indicator matrix extract maximally connected subnetworks *G*′ = (*V*′, *E*′) for which following formula evaluates to true for all nodes *v* ∈ *V*′ but *K* exceptions:

$$m-\left(\overset{m}{\underset{s=1}{\sum }}A^{v,s}\right)\leq L.$$

(Alcaraz et al., 2020, p. 183).

#### 2.1.2 Global Node Exceptions Strategy

The GloNE strategy only considers the *L* parameter. *L* describes the number of allowed case exceptions over all nodes of the extracted subnetwork *G*′. This means in an extracted subnetwork, the number of patients/samples that are not differentially regulated over all nodes must be less or equal to *L*. In contrast to INES, we do no longer consider each gene individually but have a global budget of exceptions that can be spent for maximizing a solution. Nodes which are active in most cases will thus be cheaper to add to an existing solution than nodes which are mostly not active.

The strategy can be formally defined as follows (Alcaraz et al., 2020, p. 184): Given a biological network *G* = (*V*, *E*), an indicator matrix *A* ^*n*,*m*^, *L* case exceptions and *s* being a sample from the indicator matrix extract maximally connected subnetworks *G*′ = (*V*′, *E*′) for which the following formula evaluates to true:

$$\left(|V^{\prime }|*m-\underset{v\in V^{\prime }}{\sum }\overset{m}{\underset{s=1}{\sum }}A^{v,s}\right)\leq L.$$

### 2.2 Algorithms for Network Extraction

INES and GloNE are both computationally hard problems. Three types of algorithms have been implemented to make the computation of the solutions as efficient as possible.

#### 2.2.1 Fixed Parameter Tractable

To solve the INES problem for small *K* values a fixed-parameter tractability approach for extracting exact solutions can be used. The approach applies an exact branch and bound algorithm to extract optimal subnetworks. If for a given *x*, the algorithm has a partial solution which already has *K* − *x* exception nodes, the algorithm computes the upper bound in the following steps: First it determines all possible new exception nodes that are reachable from the current subgraph in *x* steps and then considers the *n* nodes with the highest weights and adds those together. Once the upper bound is determined, the algorithm uses the two bounds in an exhaustive search to find the optimal solution (Alcaraz et al., 2012, p. 4-5, p. 8-10).

#### 2.2.2 Greedy

The Greedy approach offers an efficient way to tackle large problem instances like dense networks. The algorithms starts by adding a seed node *v* to an empty partial solution *S* = {} and then iteratively adds the adjacent node *u* to the partial solution that maximizes the following scoring function:

$f\left(S,u\right)=\sum_{r\in \left(V\left(S\right)\right)\cup \left\{u\right\}\left.\right)}\sum_{i=1}^{p}\sum_{s=1}^{m}A_{i}^{r,s}$ ; The process is repeated until no more nodes are left which could be added to gain a valid solution (Alcaraz et al., 2020, p. 184-185).

#### 2.2.3 Ant Colony Optimization

The third option is a heuristic approach based on Ant Colony Optimization called Max-Min Ant System (MMAS) (Dorigo, 2004). Given enough time this algorithm can provide an improved solution compared to the Greedy approach. The ACO algorithm starts from a seed node *v* from where you can imagine multiple ants searching for valid solutions. The ants add the next valid node based on a probability proportional to *η*(*u*)^*α*^^∗^ *τ*(*u*)^*β*^. *η*(*u*) is a heuristic value of node *u* which is proportional to the number of active cases and *τ*(*u*) is the current pheromone level of node *u*, which is proportional to the amount of ants which have previously used this vertex to create a valid solution, while the parameters *α* and *β* control the importance of *η*(*u*) and *τ*(*u*). When there is no valid node left for any ant to add, the pheromone levels are updated. First the pheromone values of all edges are decreased by an evaporation factor. Then the best solution is extracted and the pheromone levels for the nodes within this network are increased. The whole procedure is repeated until it converges to one solution (Alcaraz et al., 2020, p. 185).

### 2.3 Implementation

KPM-R, is based on two previously developed libraries: 1) the KPM-Standalone library, and 2) the KPM-Web library (List et al., 2016). The two KPM libraries allow the user to switch between a local and remote execution within the R package. The local version executes the KPM standalone jar, which is included when installing the KPM-R from GitHub; the remote version utilizes the KPM webserver to execute the method.

The communication between the standalone and R application was established using the rJava package, a low-level R to Java interface (Urbanek, 2020). In the remote execution, the RESTful API from the KPM-Web module was utilized. To access the Web-API, KPM-R uses the RCurl package to create HTTP requests (Temple Lang, 2020) and the rjson package to convert R objects into JSON ones (Couture-Beil, 2018).

#### 2.3.1 Input Data

KPM-R requires two types of input data. The first type is a biological network from which the pathways should be extracted. Some examples of networks that can be used are HPRD (Keshava Prasad et al., 2009), STRING (Szklarczyk et al., 2019), or BioGRID (Oughtred et al., 2018). These networks can either be downloaded with the help of R packages or manually from the corresponding website. A plethora of biological networks can be found and downloaded from the Network Data Exchange (Pillich et al., 2017) using the NDExR package (Auer et al., 2021). For an example on how to use NDExR and prepare the downloaded networks for KPM-R, see our vignette. KPM-R accepts a biological network in three ways: as the file path to where the network is located (in SIF format), as an igraph object, or as a network from the KPM web server, which is exclusively for remote execution and allows for selecting one of the web server’s multiple networks. Every network on the server is assigned a unique id, which can be observed using get_networks(). Once the desired ID is determined, the user can specify the graph ID with the options function, for instance, like this kpm_options(graph_id = 2).

The second type of input is one or multiple indicator matrices derived from OMICS datasets. The rows of a matrix represent the biological entities (e.g., genes), and the columns the cases/samples. In the case of a differential expression experiment a column would indicate if the genes of a certain sample (e.g., a patient) are differentially expressed compared to the control samples (e.g., the healthy individuals). An entry of “1” would indicate differential expression for a gene between a sample and the reference. All other entries have to be “0”s. There are several possibilities for a user to produce such an indicator matrix. For instance, in a differential expression analysis comparing two groups (e.g., control *vs*. diseased), we can either compute a group-wise statistic (e.g., t-test) that results in an indicator matrix with one column where each entry describes whether a gene is differentially expressed or not. However, to leverage the potential of KPM we are more interested in sample-wise statistics where one value is computed for each gene and sample pair. To compute such an indicator matrix, we propose to compute the parameters (mean *μ*_*c*_ and standard deviation *σ*_*c*_) of a normal distribution based on the control samples. For each case sample s and gene with expression *x*_*s*_, we can then compute a z-score using the formula $\frac{x_{s}-\mu_{c}}{\sigma_{c}}$ . Intuitively, this will yield a z-score that indicates how many standard distributions an expression value is away from the mean of the control group. The resulting matrix of z-scores can then be binarized using a user-defined threshold on the absolute values of the matrix. For details we refer to 2.4.

After applying the selected statistic, the user can decide which genes are differentially expressed based on the p-value and log fold change (or z-score) and whether to consider up-regulated or down-regulated genes, or both. In our vignette we give two examples on how to construct an indicator matrix. The first column of the matrix has to contain the IDs of the biological entities. The ID type of the biological entities should be equivalent to the ID type of the entities in the network. The matrices should be processed so that they do not have a header in the first row. The matrix can be passed to KPM-R as a data frame and, in the case of multiple matrices, as a list of data frames.

#### 2.3.2 Execution Parameters

Once the input data have been prepared, the user can set the execution parameters using the kpm_options() function. The general way to change an option is kpm_options([key] = [value]), where the key stands for the parameter to be changed and the value for the parameter value to be set. All parameters are case sensitive. The user can also provide multiple key-value pairs separated by a comma in one command. In total, 31 parameters can be used to adjust the execution settings to the user’s preferences. For most of the parameters, a default value is defined, which allows the execution of KPM-R with the configuration of just two parameters, *K* and *L*. The most important parameters of KPM-R can be found in Table 1. A complete list of all options and their default values can be retrieved when running the command ?kpm_options().

**TABLE 1:** KPM run options and their description.

Parameter	Description
execution	Defines the execution type of KPM-R, which can be run either “Local” *via* RestfulAPI or “Remote” *via* standalone jar.
	Default value: “Local”.
strategy	Can be either “INES” or “GLONE”. If the GloNE strategy is selected, the user does not need to set the *K* parameter.
	Default value: “GLONE”.
algorithm	The algorithm that should be used to extract the pathways. It can be set to “Greedy”, “ACO” or “Optimal”.
	Default value: “Greedy”.
use_range_k	Boolean parameter that describes whether parameter *K* should be ranged or not (see below).
	Default value: FALSE.
k_min, k_max, k_step	Numeric parameters that control the number of node exceptions allowed in a solution. If the use_range_k parameter is set to false, only k_min must be defined. Otherwise, a range must be defined with k_min and k_max defining the lower and upper boundary respectively and k_step describing the incrementation from one iteration to the next. For example, setting k_min = 4, k_max = 8 and k_step = 2 would mean that KPM will be executed with *K* = 4, *K* = 6 and *K* = 8.
	Default values: k_min = 1, k_max = 3, k_step = 1.
use_range_l	Boolean that describes whether parameter *L* should be ranged or not.
	Default value: FALSE.
l_min, l_max, l_step	Numeric parameters that control the number of case exceptions within a node. Similar to the *K* parameter, ranged values can be defined if use_range_l is set to true.
	Default values: l_min = 0, l_max = 0, l_step = 1.
link_type	When using multiple datasets, the user must specify a logical formula to combine these. The link_type parameter’s accepted values are “OR”, “AND”, or a custom formula.
	Default value: “OR”.
graph_id	ID of the network on the web server, which should be used in a remote run
negative_nodes	Character vector contains biological entities that should be considered as inactive
positive_nodes	Character vector contains biological entities that should be considered as active

### 2.4 Data Processing

Several convenient functions were implemented to make the user’s data processing workflow as easy as possible. One of them is the compute_z_score() function, which computes the genes’ z-scores in all case samples while using as background the control samples. The function receives a count matrix as input and returns a z-score matrix. The z-score of a gene *i* in a sample *j* is computed in the following way:

$$Z_{score}\left(gene\;i,sample\;j\right)=\frac{counts\;of\;gene\;i\;in\;sample\;j-mean\;of\;gene\;i\;in\;control\;samples}{standard\;deviation\;of\;gene\;i\;in\;control\;samples}$$ (1)

Another convenient function is the to_indicator_matrix() function. This function converts a p-value matrix, describing the significance of the biological entities, into an indicator matrix. The function receives two parameters as input, which are the p-value matrix and the threshold for setting active entities.

The import_graph() function allows the user to convert their graph file into an iGraph object, which is the input format required by the package. The user can choose from a variety of graph file formats, such as sif, gml, graphml, xlsx and documents with user-defined delimiters.

Furthermore, the user can utilize the export_graph() function to export pathways computed by the package. Given a pathway, the user can export the network in one of the following formats: sif, gml, graphml, xlsx, csv, igraph object or using a customer delimiter. The user can also extract only the nodes of the pathway by using the export_nodes() function.

### 2.5 Input of Single Cell RNA-Seq Data

The function sc_to_indicatormatrix() allows to generate an indicator matrix based on differential expression from single cell input data. The differential expression detection is performed by a two-part generalized linear model implemented in the MAST package which allows to address the additional complexity of scRNA-seq data and also adjustment for covariates (McDavid et al., 2021). The user has to provide single cell RNA-seq data which is normalized but not transformed, yet. The data is accepted in form of a Seurat (Satija et al., 2015), SingleCellExperiment (Amezquita et al., 2020) or SinglCellAssay (McDavid et al., 2021) object. In case of a Seurat Object the data should be in the Assay named “RNA”. For SingleCellExperiment objects the first assay is considered. The input data is log2 transformed and a hurdle model is fitted using MAST’s zlm function. This generalized linear framework can be used to jointly estimate variation from biological and technical sources, as well as the effects of interest. The function controls by default for proportion of genes expressed in a single cell. User can adjust for more complex designs by passing a formula object to the function. A likelihood ratio test is then performed for each conditioned against a user chosen reference. False discovery rate adjustment is finally performed by the Benjamini & Hochberg correction method (Benjamini and Hochberg, 1995) and data is filtered by p-value and fold change which can be chosen by the user. As the method takes into account that multiple samples can be derived from the same individual, type 1 error rate is reduced and compared to a pseudo bulk approach, a type 2 error inflation is avoided.

### 2.6 Use-Case Data

#### 2.6.1 SARS-CoV-2 Data

GEO set GSE152075 consisting of nasopharyngeal swabs from 430 individuals with SARS-CoV-2 and 54 negative controls, was downloaded using the GEOquery package in R. The downloaded samples were normalized using the TMM (Trimmed Mean of M-values) normalization method from the edgeR package (McCarthy et al., 2012). Subsequently, a differential expression analysis was carried out in which the z-scores of the genes in the case samples were calculated using the z-score function (Subsection 2.4). Three different cutoffs were used to determine the best-suited z-score for the dataset. For every applied cutoff, the average number of differentially expressed genes over all samples was computed. From these observations, an indicator matrix was created for which the z-score cutoff of 2 was taken. Together with the human BioGRID PPI network an INES run with the Greedy algorithm was performed using *L* parameter values between 20 and 220 with a step size of 20 and *K* parameter values between 2 and 20 with a step size of 2. An enrichment analysis was performed with the profile_pathway function using the pathway with the configuration *L* = 220 and *K* = 20. Significant results with the highest intersection size were further manually inspected.

#### 2.6.2 Tabula Muris Senis Data

Processed scRNA-seq data was retrieved from Figshare https://doi.org/10.608 4/m9.figshare.12 827 615.v3 by downloading the rds_by_tissue.14.zip file. The droplet.normalized.Limb_Muscle.rds file was then selected and was filtered for “mesenchmymal stem cells” and “mesenchmymal satellite stem cells”. Mice aged 1 and 3 months were considered as young control cases while mice aged 18, 21, 24, and 30 months were treated as old. Differential expression between young and old mice was performed based on a linear mixed effect model from the MAST package while controlling for sex and number of genes per cell. Each mouse of the old age group was treated as a case and compared against all young mice, resulting in 12 in comparisons. Genes below a Benjamini-Hochberg FDR corrected p-value of 0.05 were treated as differently expressed and considered as active in the indicator matrix. The *Mus musculus* BioGRID was chosen as a biological network and filtered for genes which were expressed in the mouse tissue. Given the input data, a grid run with *L* values between 1 and 6 and *K* values from 0 to 10 with the INES strategy and the greedy algorithm was performed.

## 3 Results

### 3.1 Workflow

**FIGURE 1:**
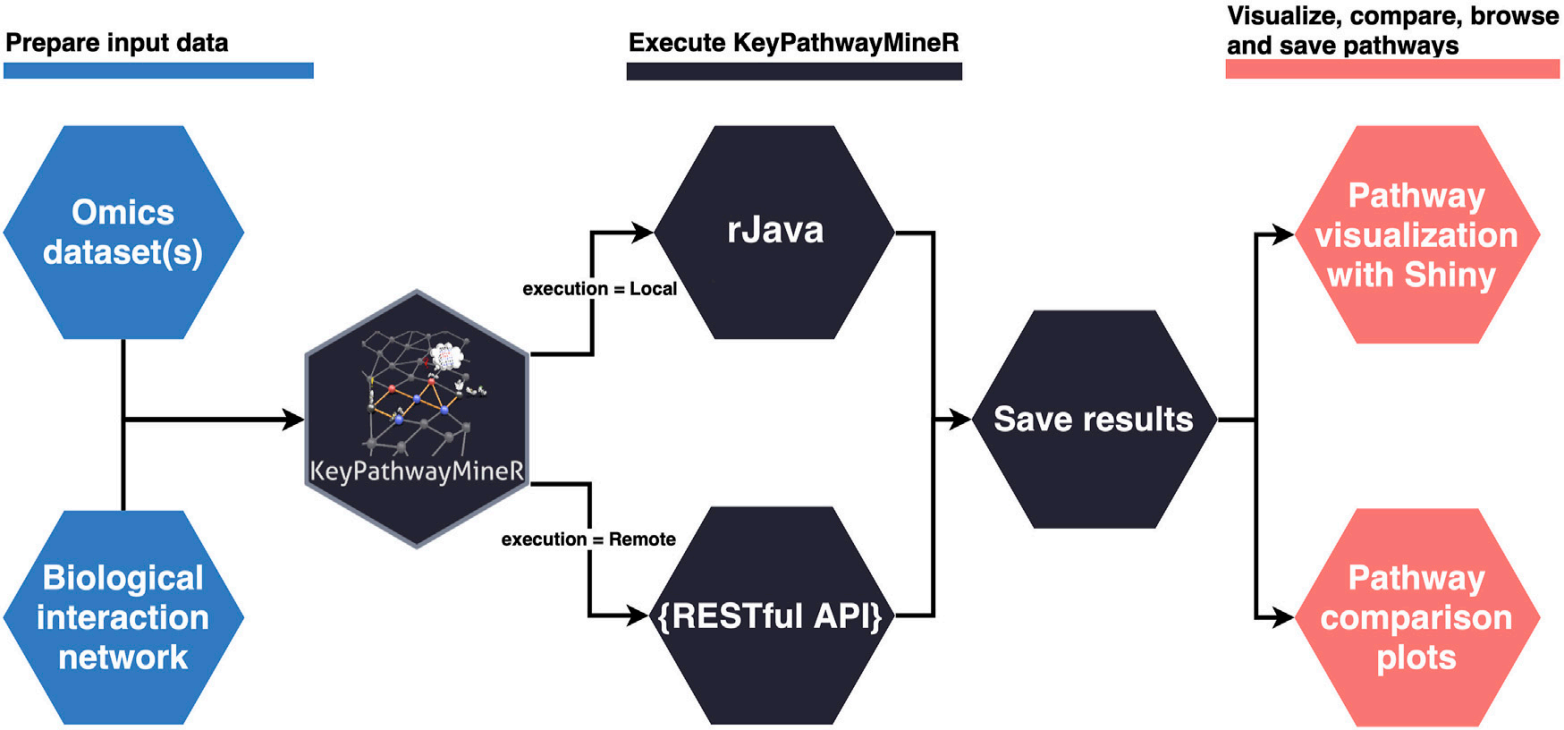
Typical workflow of KPM-R.

The lack of a user-friendly solution for *de novo* enrichment in the R ecosystem motivated us to develop KPM-R. The typical workflow when using KPM-R can be divided into three steps, also depicted in Figure 1:

#### 1. Data preparation

One or multiple OMICS datasets and a biological network are loaded into R. The biological network has to be either saved as a file in one of the supported graph formats (see 2.4) or has to come as an igraph object. Using KPM-R’s internal function retrieve_biogrid() various biological networks from BioGRID can easily be loaded in the proper format. The user can select the gene identifier and the organism which match the OMICS data. OMICS datasets must be transformed into indicator matrices if they are not already in this form. Each matrix entry is either a ‘1’ indicating an active case in a node or ‘0’ otherwise. For example in a gene expression dataset a ‘1’ would represents a differentially expressed gene or in bisulfite sequencing data a differentially methylated promotor of a gene. Optionally the user can also incorporate previous knowledge and provide positive and negative lists defining genes which are always considered active or that are ignored. For the generation of indicator matrices KPM-R comes with two convenient functions. The to_indicator_matrix() function converts a p-value matrix, describing the significance of the biological entities, into an indicator matrix. The sc_to_indicator_matrix() functions allows to take input of single cell RNA-seq data in various common object formats.

#### 2. Execution of KPM-R

Once the datasets are prepared, the run’s execution parameters must be set. Most importantly, the user has to decide to either use the Individual Node Exceptions (INES) strategy, or the Global Node Exceptions (GloNE) strategy for the discovery of meaningful pathways (Alcaraz et al., 2020). Both strategies are computationally hard problems and subnetwork extraction can either be based on a greedy approach, a heuristic based on Ant Colony Optimization or, for INES, even by calculation of the exact solution for small *K* values using a fixed-parameter tractable algorithm. The parameters for a run can be set with the kpm_options() function, where the user has the possibility to define ranged parameter values instead of unique ones. The optimal values for *K* and *L* depend on the dataset, therefore the function allows several iterations with different configurations of *K* and *L* values to be performed in one run. Generally, larger *K* values result in larger networks. When choosing the parameter the user has to be aware of the underlying hypothesis of his experiment. For instance, the up to *K* exception nodes that correspond to genes that are not differentially expressed may still be disease-relevant due to mutations. Alternatively, they may pose promising drug targets as they are of central importance to disease-associated genes. Choosing large *K* values, however, increasingly leads to the incorporation of hub nodes which connect to distant parts of the network and thus different pathways which may not be functionally related. For the choice of the *L* parameter, the user should look at the ratio of the number of samples and a possible *L* value. For example, an *L* value of 10 in a study with 20 cases would mean that for a given node, at most 50% of the cases may not be differentially expressed. Users need to select a suitable value here which fits their expectation but they can also perform a grid search to conveniently explore different *L* values. The parameters of a run can be globally set and, subsequently, the kpm() function can be used to execute the program given a biological network and a list of indicator matrices as input. Once the execution of the program is completed, the results are saved in a unified manner, and an export function allows the user to easily extract the pathways as igraph objects or in several graph formats.

#### 3. Pathway visualization

**FIGURE 2:**
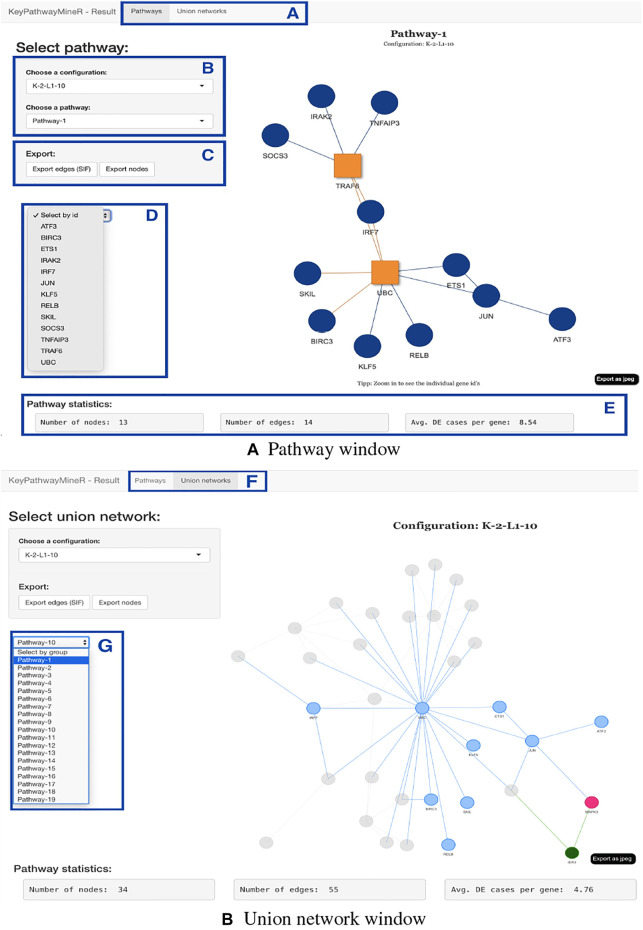
Shiny app for visualizing, browsing, and saving pathways from a result object. The displayed subnetworks were extracted from SARS-CoV-2 gene expression data. **(A,F)** The user can switch between pathway and union network view. **(B)** Panel to select which parameter configuration and subnetwork to visualize. **(C)** Export buttons that allow the extraction of the current pathway as edges or nodes. **(D)** The user can select a gene from the network for closer inspection. **(E)** For every network, statistics are displayed, which provide information on the number of nodes, edges, and the average number of active cases per node. **(G)** In the union network view, the selection panel allows selecting pathways to examine from which subnetwork the nodes originate. When using INES to run KPM, the exception nodes are marked as red squares, as shown in the pathway view.

**FIGURE 3:**
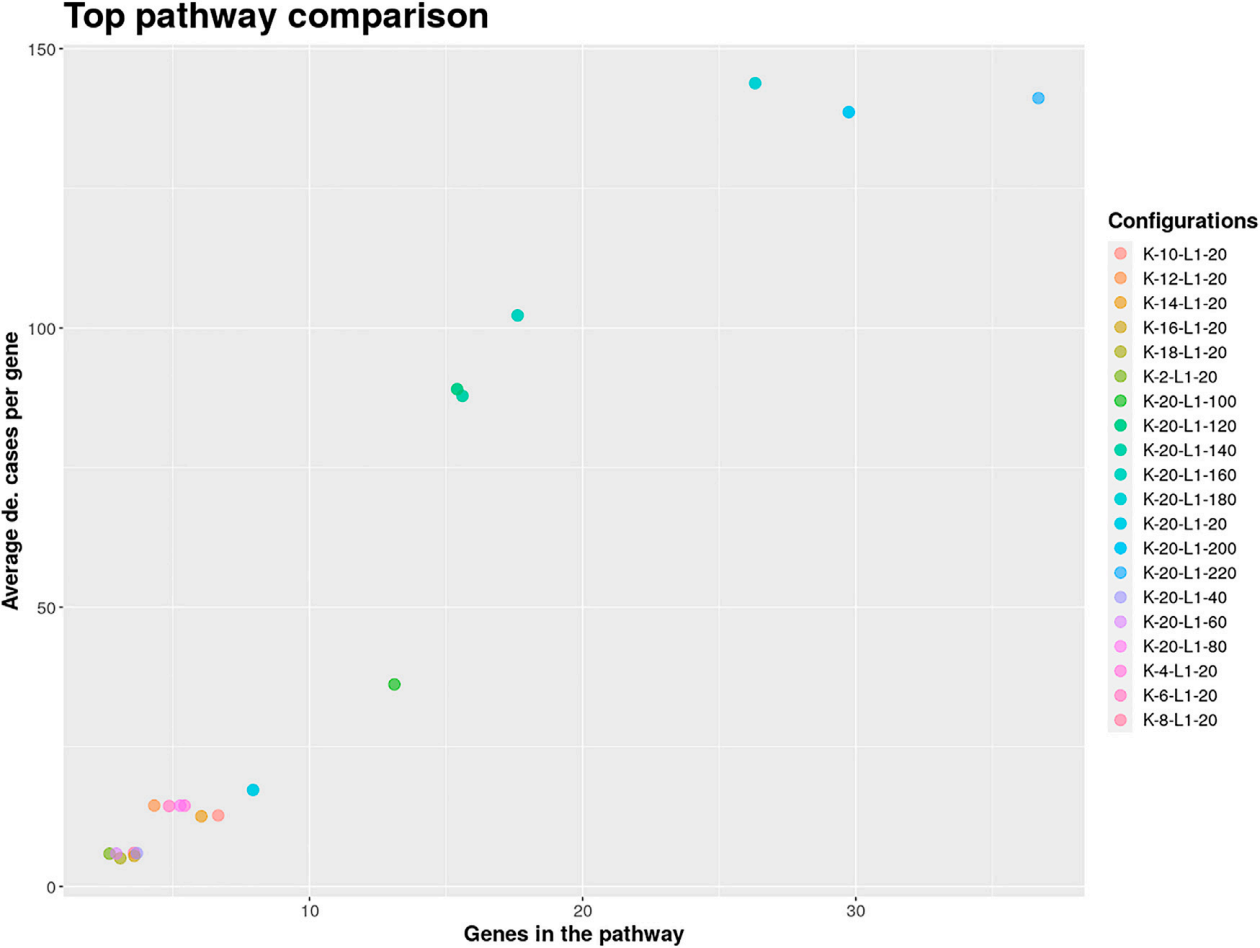
Pathway comparison plots can be utilized to find the optimal pathway in the extracted solution. The shown pathways were extracted from the analyzed GEO SARS-CoV-2 dataset and used to limit the further exploration to configurations with at least 100 on average differentially expressed genes per case.

The result object obtained from the run can then be used to visualize, browse and save pathways with an interactive web app which was developed using Shiny (Chang et al., 2021) and visNetwork (Almende et al., 2019). The function visualize_results() allows the user to browse through the pathways and also to save them as an image or text file. A node in a pathway can be in two states, either in an exception state where the number of inactive cases for this node is above the *L* value or in a significant state in which the number of inactive cases is at most *L*. Nodes in an exception state are symbolized by an orange square and nodes in a significant state are represented by a blue circle. This does not apply to the GLONE strategy, which does not contain exception nodes and therefore only contains circles. If the nodes in the subnetworks are genes, the user can click on them to be forwarded to the corresponding gene entry on the NCBI website (Benson et al., 1990). In Figure 2, the interface and the single components of the Shiny app are presented and described. Using the function pathway_comparison_plots(), two plots are generated: one comparing the top pathways and one comparing the union networks (all pathways of a configuration merged) of every parameter-configuration (see Figure 3 for an example). Specifically, the plots compare all parameter configurations by plotting the average active cases per node (e.g., the sum of differentially expressed cases for a certain gene) against the number of nodes in a pathway. These plots serve as an aid to users to select the best pathways for in detail exploration and further downstream analysis. When the user has found an interesting pathway, the shiny app allows to easily download this network in SIF format, simply by clicking the button “Export edges (SIF)”. Finally, the user can also directly perform downstream analysis with the profile_network() function and easily visualize its result with gprofiler2’s plot functions (Kolberg et al., 2020).

### 3.2 Use Cases

Two application cases were selected to evaluate the usability and features of KPM-R. The use-cases aimed to demonstrate the benefits of conducting an analysis with KPM-R, the integration of KPM-R with Bioconductor, and the power of KPM-R in finding meaningful results. The majority of the analysis steps, from the data acquisition to the further downstream analysis of the results, were conducted within the R framework.

#### 3.2.1 SARS-CoV-2 Infection

The COVID-19 pandemic has confronted our society with major challenges. For this reason, research into understanding and combating the infectious agent, SARS-CoV-2, is important. The amount of publicly available data generated in context to SARS-CoV-2 is continuously increasing and can be used for further in depth analysis. Here, we used a large bulk RNA sequencing dataset of nasopharyngeal swabs from 430 SARS-CoV-2 infected individuals and 54 negative control cases. The raw counts were directly downloaded into R using the GEOquery package (Davis and Meltzer, 2007b) and subsequently normalized using edgeR (McCarthy et al., 2012). Mean and standard deviation was calculated for every gene using the control group’s gene counts as reference. Genes with a z-scores below −2 and above 2 were considered as differentially expressed. With the generated indicator matrix and the BioGRID as a biological network, KPM-R was executed in a grid run for *L* values between 20 and 220 with step size 20 and *K* values between 2 and 20 with step size 2, using the INES strategy and the Greedy algorithm. Intuitively, this means that the genes in the extracted solutions will be in at most 51% (220/430) of the studies inactive and that an extracted solution will have at most 20 exception genes.

**FIGURE 4:**
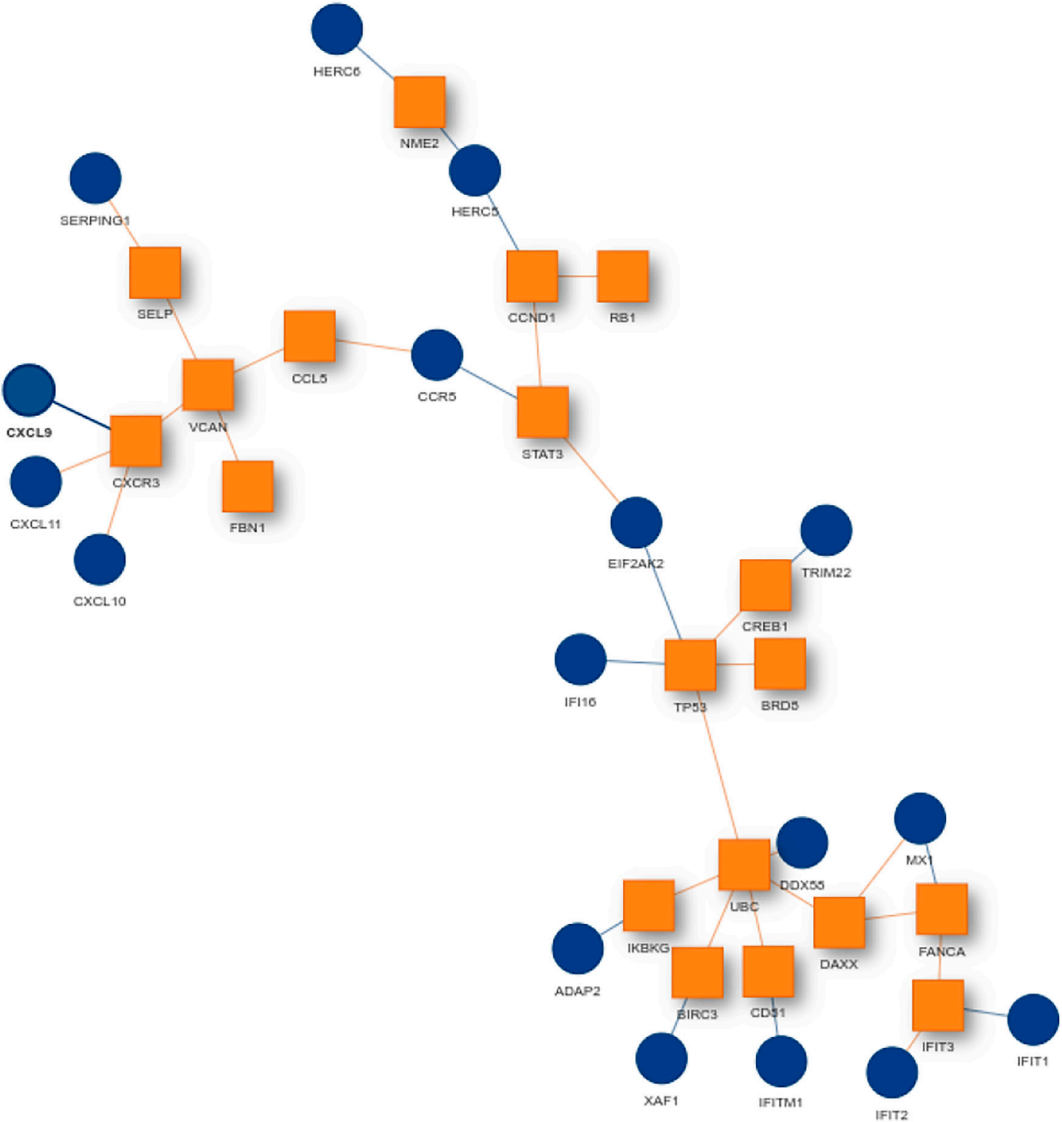
SARS-CoV-2 network from the configuration *L* = 220 and *K* = 20. Many genes of the network are known to be important players in the human immune response. Exception nodes are visualized as orange squares and significant nodes as blue circles.

**FIGURE 5:**
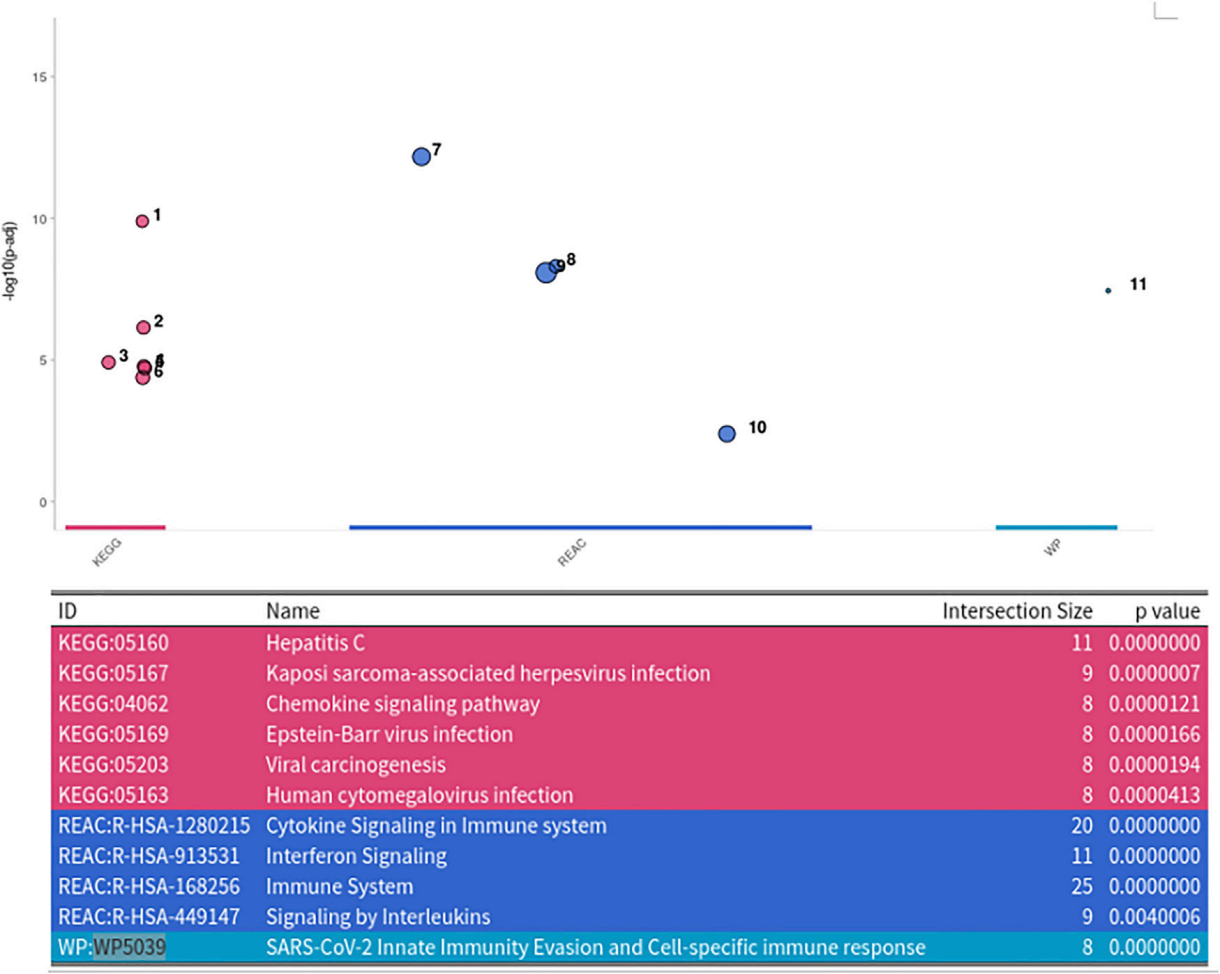
Enrichment Analysis of extracted SARS-CoV-2 network. The network was tested for enrichment within the KEGG, Reactome and Wikipathways data bases.

For finding the most promising pathways a comparison plot was generated (Figure 3). Networks with on average more than 100 differential expressed cases were examined. Exploring these results we find a network with many known and recently described key players of Covid-19 (Figure 4). Looking at the leaf nodes, we see many genes which are an important part of human immune response like interferon induced genes IFIT1/2 and IFI16 or chemokine ligands like CCL5/9/10/11. Inspecting the central nodes we find the antiviral kinase EIF2AK2, also an important actor in the innate immune response (Ishaq and Natarajan, 2020). Its direct neighbor TP53 is known to facilitate EIF2AK2’s expression and also acts as host antiviral factor by itself. Recently, SARS-CoV-2’s papain-like protease of the nonstructural protein 3 was shown to downregulate TP53 at the protein level by manipulations of it’s ubiqutiniation and thereby facilitating viral replication (Ma-Lauer et al., 2016). Although TP53 is an exception node and hence does not show differential abundance on the transcript level, SARS-CoV-2’s induced TP53 degradation might contribute to EIF2AK2’s downregulation and consequently further weaken the anti viral defense. EIF2AK2 is also linked to STAT3, another important regulator of the immune response. In many Covid-19 patients STAT3 is hyperactivated which is associated with cytokine release syndrome and acute lung injury (Matsuyama et al., 2020). Moreover, STAT3 is described to inhibit PKR’s activity and hence its overexpression potentially leads to another weakening of the viral defense mechanisms (Niso-Santano et al., 2013). STAT3 is an exception node and is only differentially expressed in 6% of the patients, therefore this interaction might only be relevant for a subtype of patients. Unfortunately, we lack the metadata to see if the differential STAT3 expression could be linked to features like the severity of the infection. STAT3’s function could also be affected on the protein level by adjacent nodes. It’s a strength of KPM that the results can include genes which might not be differentially expressed on the transcript level but still be part of a biological cascade. The network also shows an interaction between STAT3 and its adjacent node CCR5 which has STAT3 binding sites in its promoter (Yoo et al., 2014). CCR5 has already been part of a initial clinical trials which showed that its inhibition can decrease inflammatory cytokines in Covid-19 patients (Patterson et al., 2021). To make sure that we were not exploring a previously described pathway, we performed functional enrichment analysis with our integrated pathway enrichment function. Here we checked for enrichment within KEGG, Reactome and WikiPathways and filtered for the significant hits with the highest intersection size (number of genes of our extracted network found within a certain pathway) (Figure 5). We found that various genes of our network are also part of known pathways in viral infections including SARS-CoV-2. However, at most 30% of the genes detected by KPM were part of one of these networks. The highest overlap was found with the terms “Immune System” and “Cytokine Signaling in Immune system” where respectively 55 and 70% of the genes in our network could be found. Still our network contains around 30% genes which were not described in these pathways, indicating that we could indeed have found a pathway which was not described previously. We leave further exploration and interpretation of the data to the experts in the field. We hope that examination of the networks generated by KPM-R might even offer potential clues for the experts on how to infer with one of these processes.

#### 3.2.2 Aging Tissue From Tabula Muris Senis

Muscle tissue in mammals has various functions including mobility, body temperature regulation, energy storage and support of soft tissue. However, muscle mass and function decline in mammals with age, a condition named sarcopenia. Understandably, the loss of muscle mass and function is associated with numerous morbidities and mortalities and sarcopenia has a great impact on healthcare costs (Larsson et al., 2019). Rodents and humans suffer from a decline in muscle tissue with aging in a comparable way which makes them a good model organism for the study of muscle atrophy (Baek et al., 2020).

The *Tabula Muris Senis*, or ‘Mouse Ageing Cell Atlas’ is a great resource for transcriptomic data of the aging mouse tissue (Almanzar et al., 2020). The atlas consists of single-cell RNA sequencing data of more than 350,000 cells from male and female mice tissues belonging to six age groups, ranging from 1 month to 30 months. Mice at the age of 1 month can be compared to humans at a early childhood, while 30 months old mice are the equivalent of a human centenarian (Almanzar et al., 2020). We aimed to use KPM-R to identify differentially regulated pathways in the aging tissue. Therefore, we used single cell data from the limb muscle and focused on the mesenchymal stem (MSC) and satellite cells. In the muscle tissue, MSCs can give rise to satellite cells. While these cells which are named after their satellite position on the myofibre, differentiate into myoblasts and are responsible for muscle repair and growth. Mice age 1 and 3 months were treated as young control cases and were compared to the remaining time points. We used our internal functions based on MAST package’s linear mixed effect model for finding differentially expressed genes between the young and old mice. Together with the *Mus musculus* interaction network from BioGRID an INES run with *L* values between 0 and 6 and *K* values between 0 and 10 was performed.

**FIGURE 6:**
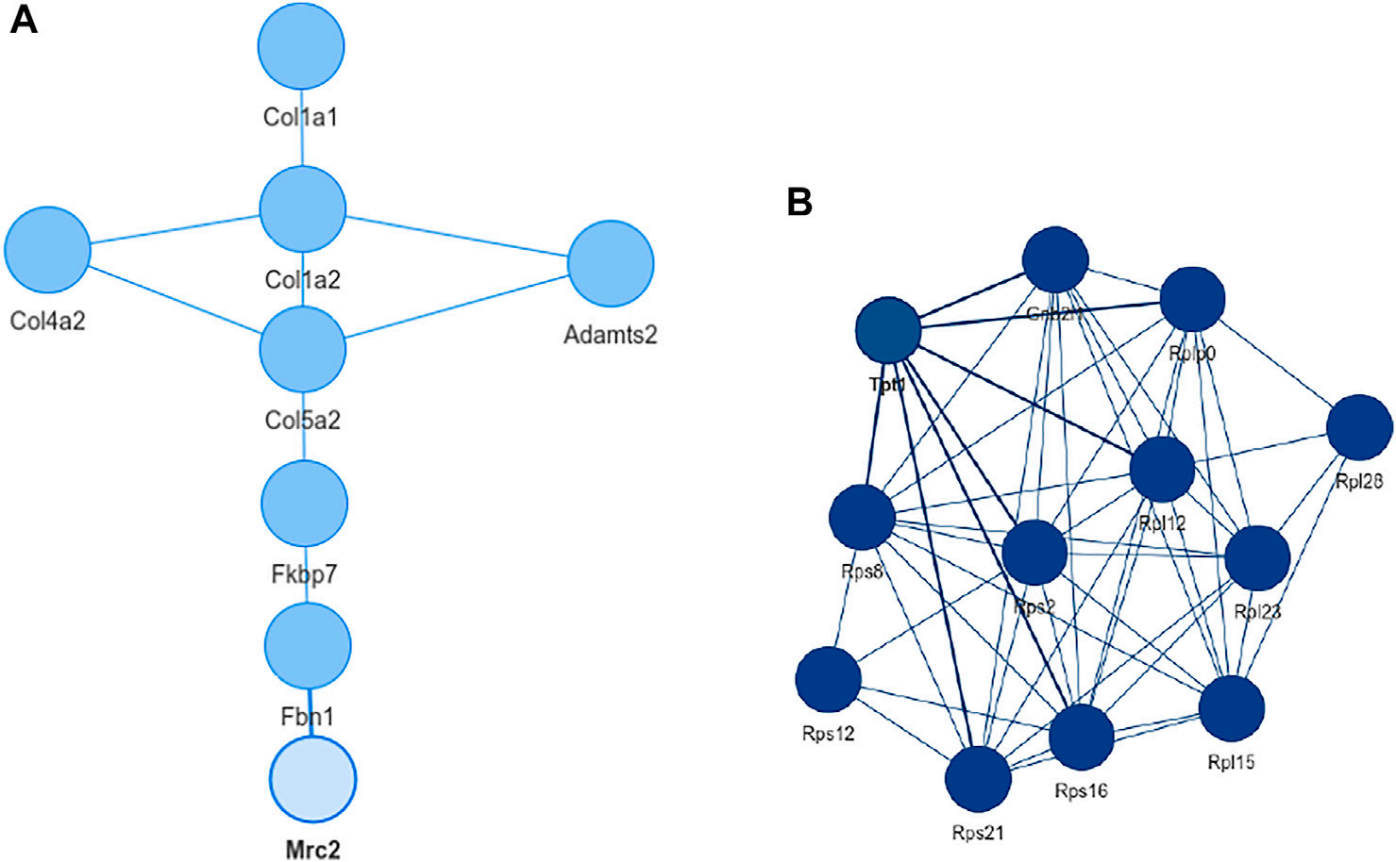
Extracted networks from single cell data of murina limb muscle. **(A)** Network with the configuration *L* = 0 and *K* = 0 based on mesenchymal stem cells from the limb muscle. The genes consist mainly of extracellular matrix proteins and are differentially expressed in all old mice. **(B)** Network of mesenchymal satellite stem cells with *K* = 0 and *L* = 4. With the exception Tpt1 and Gnbl2l1, all nodes are ribosomal proteins.

**FIGURE 7:**
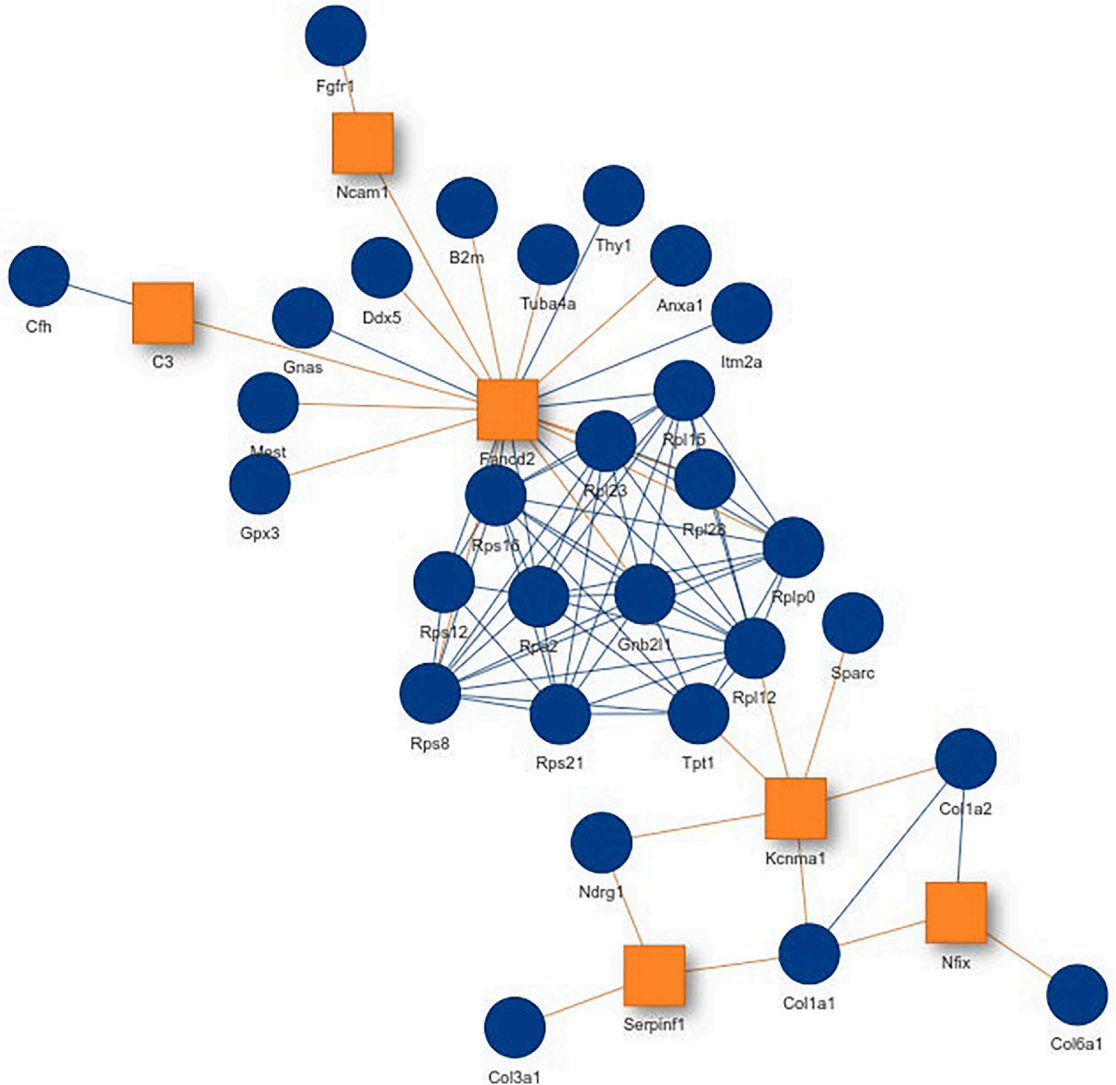
Network from single cell data of satellite stem cells from the murine limb muscle with the configuration *K* = 6 and *L* = 4. Exception nodes are visualized as orange squares and significant nodes as blue circles.

Inspecting the MSC results we could extract a small network where each of the genes is differentially expressed in all aging mice (Figure 6A). It consists of extracellular matrix proteins including members of the collagen family like Col1a1/2, fibrillin-1 and proteins potentially involved in the processing of these proteins like the metalloprotese Adamts2. Collagen is the most abundant protein in mammals and depending on the tissue it consists of a variable mixture of different collagen proteins. In muscle tissue it serves as the major component of the endomysium which ensheats each muscle fiber. During aging the skeletal muscle’s connective tissue compartment is known to show significant changes (Goldspink et al., 1994; Evans et al., 1995). The computed network can be seen as a proof of KPM-R’s ability to find biological meaningful interactions among the single cell dataset. Taking a further look at the networks of the satellite cells we see various networks which include a highly connected networks of ribosomal proteins (Figure 6B). It was recently shown that dozens of proteins involved in the ribosome biogenesis are down regulated with age which is consistent with age related decline in protein synthesis (Anisimova et al., 2020). Enrichment analysis showed that this network is part of already annotated pathways. However, we found these networks of ribosomal proteins also being connected to various other proteins (Figure 7) which might make them worth exploring for the specialists in the field.

## 4 Conclusion

KeyPathwayMineR is an established tool in the field of *de novo* pathway enrichment and was so far available as a Java stand alone tool, a web-server version and a Cytoscape plugin. Here we have created a package of KPM in the R programming environment which has a rich community of biological scientist who appreciate easily understandable workflows. Due to KPM-R’s interoperability with a plethora of existing R packages, which can be found on Bioconductor, CRAN, or GitHub, KPM-R allows access to a large number of datasets, networks, data processing and down-stream analysis methods which can now be conveniently integrated into fully reproducible analysis workflows.

We demonstrate the packages abilities by applying KPM-R on a large bulk RNA-seq dataset of nasopharyngeal swabs from SARS-CoV-2 infected individuals. The data of this study was downloaded and prepared in R with well known packages like GEOquery and edgeR. After performing a grid run with KPM-R, the pathway-comparison-plots function allowed us to easily find the most promising networks. The inspected SARS-CoV-2 network contained many already described key player in Covid-19 disease like STAT5, EIF2AK2 or various chemokines. We also found TP53 as a common interaction partner in the network and a transcription factor of its neighbor node EIF2AK2. Although TP53 did not show differential abundance at the transcript level it’s known to be downregulated by SARS-CoV-2 at the protein level. Hence we show that exception nodes can still be meaningful interaction partners and KPM-R coupled with domain knowledge allows to find potentially interesting pathways.

During the last years, single cell RNA sequencing became increasingly popular and is slowly replacing bulk RNA sequencing as the major method to study transcript abundances. In KPM-R we also implemented functions which make it easy to work with single cell RNA sequencing data. Using single cell data of the *Tabula Muris Senis* Atlas, we show that KPM-R can find potentially interesting pathways in the aging murine muscle tissue. The mesenchymal stem cells showed differentially expressed networks containing extracellular matrix proteins and in satellite cells we saw densely connected nodes of ribosomal proteins. Both remodelling of the skeletal muscle’s connective tissue and ribosome biogenesis are processes already previously linked to aging. We leave these networks to be further explored by the experts in the field.

In conclusion, KPM-R extends the features and usability of existing versions of KPM by leveraging the power, flexibility and versatility of R, thereby providing R users with various functionalities for performing data preparation, *de novo* pathway enrichment and visualization.

## Data Availability

Publicly available datasets were analyzed in this study. This data can be found here: The datasets for this study were downloaded from the Gene Expression Omnibus (GSE152075) and from figshare (https://doi.org/10.6084/m9.figshare.12827615.v3).
